# Incidence and timing of new-onset atrial fibrillation after transcatheter aortic valve implantation: 14-day electrocardiographic monitoring

**DOI:** 10.1016/j.hroo.2026.06.019

**Published:** 2026-07-04

**Authors:** Arianna Sofia Bettelini, Céline Moser, Fabian Jordan, Thorald Stolte, Reto Stump, Thomas Nestelberger, Felix Mahfoud, Michael Kühne, Christian Sticherling, Patrick Badertscher

**Affiliations:** 1 Department of Cardiology, University Hospital Basel, Basel, Switzerland; 2 Cardiovascular Research Institute Basel, University Hospital Basel, Basel, Switzerland

**Keywords:** New onset atrial fibrillation, Cardiac rhythm monitoring, Transcatheter aortic valve implantation, Anticoagulation, Atrial fibrillation, Atrial fibrillation burden

**Figure:**
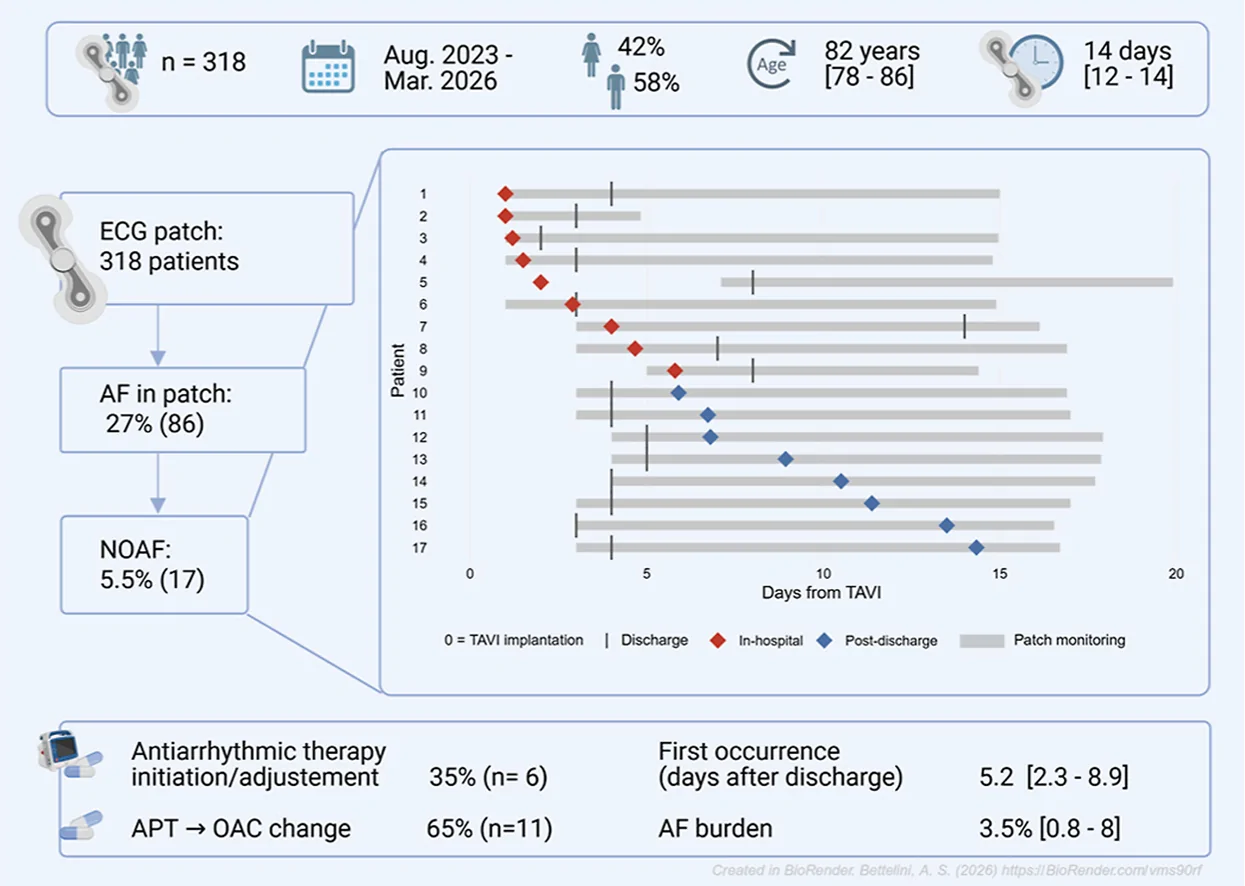

**Key Findings**

▪ Ambulatory electrocardiogram (ECG) monitoring detected new-onset atrial fibrillation (NOAF) in 5.5% of patients after transcatheter aortic valve implantation (TAVI) procedure, the majority of whom were at high thromboembolic risk.
▪ A substantial proportion of NOAF cases were detected only after hospital discharge through ambulatory ECG monitoring.
▪ Patients with NOAF were predominantly asymptomatic, had low AF burden, and paroxysmal AF.
▪ Detection of NOAF frequently resulted in initiation of oral anticoagulation and changes in rhythm-management strategies.

Ambulatory long-term electrocardiogram (ECG) monitoring after transcatheter aortic valve implantation (TAVI) allows detection of subclinical arrhythmias that may otherwise go unrecognized. Although post-procedural conduction disturbances are extensively studied, the incidence and timing of atrial tachyarrhythmias, particularly after hospital discharge, are underexplored. New-onset atrial fibrillation (NOAF) after TAVI has been associated with an increased risk of stroke and adverse clinical outcomes, including all-cause mortality.^1^ However, its temporal distribution during the in-hospital phase vs early post-discharge period remains insufficiently characterized. This gap may have important implications for rhythm surveillance and postprocedural management.

Therefore, the aim of this study was to determine the incidence, temporal distribution, and management implications of NOAF after TAVI.

Adult patients undergoing TAVI at a Swiss tertiary center were prospectively enrolled and underwent ambulatory rhythm monitoring using commercially available single-lead 14-day ECG patches applied before discharge. Patients with pre-existing pacemakers and those undergoing pacemaker implantation were excluded. ECG recordings were initially analyzed using manufacturer-provided automated algorithms and subsequently adjudicated by 2 experienced electrophysiologists (RS and PB). NOAF was defined as AF lasting ≥30 seconds in patients without a history of AF. The study was approved by the Cantonal Ethics Committee and conducted in accordance with the Declaration of Helsinki. All patients provided written informed consent.

From August 2023 to March 2026, a total of 318 patients underwent ambulatory ECG monitoring following TAVI (median age: 82 years, interquartile range [IQR], 78–86 years, 42% female). Monitoring started a median of 3 days (IQR, 2–4 days) after TAVI and continued for 13.5 days (IQR, 12–14 days). AF was detected in 86 patients (27%), including 17 patients (5.5%) with NOAF. Nearly half of NOAF cases (47%) were first identified after discharge, with the first episode occurring a median of 5.2 days later. NOAF episodes were predominantly paroxysmal (88%), asymptomatic, and of low burden (median AF burden 3.5%). The median longest AF episode duration was 223 minutes, and 88% of patients had episodes lasting ≥6 minutes.

Patients with NOAF had a high thromboembolic risk, with 94% having a CHA_2_DS_2_-VA score of ≥2 (median score 4 [IQR, 3–5]). Detection of NOAF resulted in initiation of oral anticoagulation (OAC) in 11 patients (65%), including 3 cases identified exclusively through ambulatory monitoring. At 30-day follow-up, 13 patients (77%) were receiving OAC, while 2 patients (12%) remained on antiplatelet therapy and 2 patients (12%) received combination therapy. Antiarrhythmic management was modified in 6 patients (35%).

The observed NOAF incidence of 5.5% is consistent with prior studies, including meta-analyses and studies using extended ambulatory monitoring, reporting rates of approximately 7%–10%.^2^^,^^3^ Importantly, nearly half of NOAF cases in this cohort were detected only after hospital discharge, suggesting that reliance on in-hospital monitoring may substantially underestimate the true incidence of NOAF after TAVI. The median interval of approximately 5 days from discharge to first detected AF episode highlights an early post-discharge period that is not captured by standard in-hospital monitoring strategies.

AF episodes were predominantly paroxysmal, low burden, and largely asymptomatic. These findings contribute to the ongoing debate regarding the clinical relevance and optimal management of subclinical AF after TAVI. In this cohort, NOAF detection frequently led to initiation of OAC, including in patients identified solely through ambulatory monitoring, underscoring the potential impact of extended rhythm surveillance on clinical decision-making. Concurrently, variability in antiarrhythmic management reflects persistent uncertainty regarding optimal treatment strategies for low-burden and asymptomatic AF.^4^^,^^5^

Study limitations include exclusion of patients with pacemakers, possible underdetection of early in-hospital arrhythmias, and the limited number of NOAF cases, which limit the precision of subgroup analyses.

In conclusion, a substantial proportion of NOAF cases after TAVI is first detected after hospital discharge and is predominantly asymptomatic. Extended ambulatory ECG monitoring increases detection rates of NOAF and may influence antithrombotic and rhythm-management decisions. These findings support a potential role for targeted post-discharge rhythm monitoring in selected high-risk patients undergoing TAVI, while the clinical implications of low-burden subclinical AF remain to be defined.

## Disclosures

Patrick Badertscher has received research funding from the “University of Basel,” the “Stiftung für Herzschrittmacher und Elektrophysiologie,” the “Freiwillige Akademische Gesellschaft Basel,” the “Swiss Heart Foundation,” and Johnson&Johnson, and reports personal fees from BMS, Boston Scientific, and Abbott, all outside the submitted work. Christian Sticherling: Member of Medtronic Advisory Board Europe and Boston Scientific Advisory Board Europe, received educational grants from Biosense Webster and Biotronik and a research grant from the European Union’s FP7 program and Biosense Webster and lecture and consulting fees from Abbott, Medtronic, Biosense Webster, Boston Scientific, Microport, and Biotronik, all outside the submitted work. Michael Kühne reports grants from the Swiss National Science Foundation (Grant numbers 33CS30_148474, 33CS30_177520, 32473B_176178, 32003B_197524, and 33IC30_229733), the Swiss Heart Foundation, the Foundation for Cardiovascular Research Basel and the University of Basel, grants from Abbott, Bayer, BMS, Boston Scientific, Daiichi Sankyo, Medtronic, Pfizer, and Roche. Royalties from Springer (ECG book), all outside the submitted work. Felix Mahfoud has been supported by Deutsche Forschungsgemeinschaft (SFB TRR219, Project-ID 322900939) and Deutsche Herzstiftung. FM’s previous institution, Saarland University, received scientific support from Ablative Solutions, Medtronic, and ReCor Medical. Until May, 2024, FM has received speaker honoraria or consulting fees from Ablative Solutions, AstraZeneca, Inari, Medtronic, Merck, Novartis, Philips, and ReCor Medical. Thomas Nestelberger has received research support from the Swiss National Science Foundation (P400PM_191037/1), the Prof. Dr. Max Cloëtta Foundation, the Margarete und Walter Lichtenstein-Stiftung (3MS1038), the Freiwillige Akademische Gesellschaft Basel, the Stiftung kardiovaskuläre Forschung Basel, the University of Basel, the University Hospital Basel as well as speaker/consulting honoraria or research support from Edwards Lifesciences, Pronova Medical, Meril, Boston Scientific, Medtronic, Abbott, Beckman Coulter, Bayer, Ortho Clinical Diagnostics and Orion Pharma, outside the submitted work. Thorald Stolte has been supported through the Swiss National MD-PhD Fellowship (MD-PhD 34/25) and University Hospital Basel. The remaining authors have no conflicts of interests to declare.
